# Targeting cancer with delphinidin: from molecular insights to therapeutic implications

**DOI:** 10.3389/fphar.2026.1778570

**Published:** 2026-03-25

**Authors:** Yuhong Xu, Bingqian Jin, Pingping Yin, Kaiwen Cheng, Qianqian Li, Haigang Ding

**Affiliations:** 1 Department of Gynecology, Shaoxing People’s Hospital (School of Medicine, Shaoxing University), Shaoxing, Zhejiang, China; 2 The First Affiliated Hospital of Shaoxing University, Shaoxing, Zhejiang, China; 3 Department of Medical Research Center, Shaoxing People’s Hospital (School of Medicine, Shaoxing University), Shaoxing, Zhejiang, China

**Keywords:** cancer, delphinidin, mechanism, pharmacology, review

## Abstract

Delphinidin is a natural anthocyanidin abundant in various fruits, vegetables, and flowers. It has garnered significant attention due to its potent antioxidant activity and extensive anticancer potential. This review systematically elaborates on its chemical structure, biosynthetic pathways, and therapeutic roles, with a focus on its molecular mechanisms, cancer-specific effects, and clinical challenges. Mechanistically, delphinidin exerts anticancer effects through multiple pathways, including anti-proliferative activity, promotion of apoptosis, regulation of autophagy, inhibition of migration and invasion, suppression of angiogenesis, modulation of the immune microenvironment, and chemosensitization. These multi-target actions contribute to its pronounced tumor-suppressive effects in a broad spectrum of cancers, including but not limited to breast, lung, liver, colorectal, prostate, and ovarian malignancies. Despite its promising preclinical efficacy, the clinical translation of delphinidin is primarily hindered by its low oral bioavailability and poor stability. Emerging strategies such as nano-delivery systems and structural modifications are being actively explored to overcome these limitations. In summary, as a multi-targeted, low-toxicity natural compound, delphinidin holds broad application prospects in cancer prevention, treatment, and combination therapy, provided that its pharmacological challenges can be successfully addressed.

## 1 Introduction

Cancer represents a significant threat to global health and mortality as a major and complex disease. According to the latest estimate by the International Agency for Research on Cancer, nearly 20 million new cancer cases and 9.7 million cancer-related deaths are expected worldwide in 2022. Breast cancer in women and lung cancer in men are projected to be the most prevalent types respectively (Bray et al., 2024). It is predicted that there will be over 35 million new cases by 2050, highlighting an exceptionally severe challenge for global cancer prevention and control (Bray et al., 2024). Despite the revolutionary progress brought by targeted drugs and immune checkpoint inhibitors in cancer treatment, the overall prognosis of cancer patients is still not ideal (Zafar et al., 2024). Therefore, the development of new safe and low-toxicity anticancer drugs remains a hot topic of concern for global scientific researchers.

**FIGURE 1:**
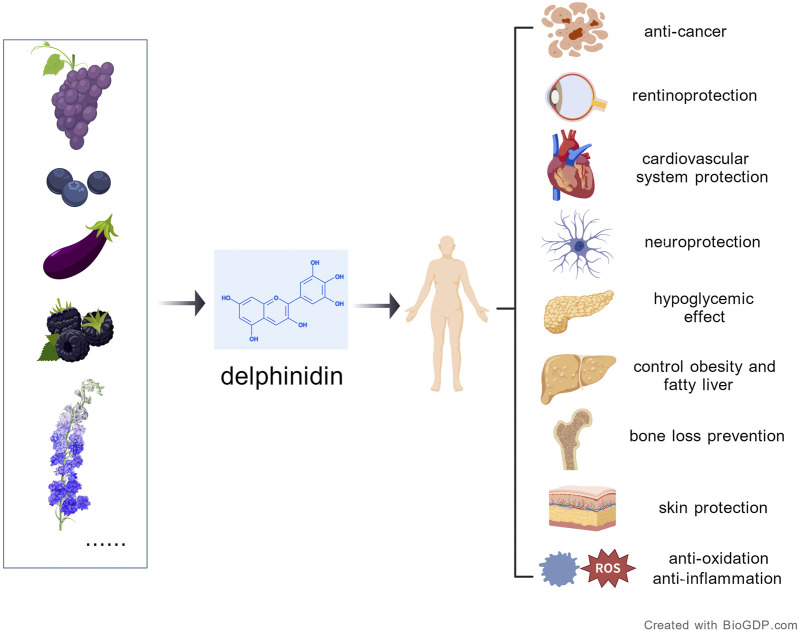
The origins of delphinidin and its benefits to human health.

Natural compounds, especially flavonoids, have emerged as promising anticancer agents due to their diverse biological activities and low toxicity to healthy tissues (Khan et al., 2021; Wang et al., 2023). As water-soluble flavonoids present in vegetables, fruits, and other plants, anthocyanins demonstrate strong anti-inflammatory, antioxidant, anti-tumor, vision-protective, and blood-glucose-lowering effects (Sadowska-Bartosz and Bartosz, 2024; Yücetepe et al., 2024). So far, more than 20 natural anthocyanin aglycones have been identified, the most common six of which are cyanidin, delphinidin, pelargonidin, peonidin, petunidin, and malvidin (Sinopoli et al., 2019). Delphinidin is present in various brightly colored fruits, such as blueberries and blackberries. Additionally, it can be found in vegetables, flowers, and dietary supplements (Yun et al., 2009). As an anthocyanidin, delphinidin contains the highest number of hydroxyl groups among its class, exhibits potent antioxidant capacity attributable to this distinctive polyhydroxylated structure, which underlies its significant biological functions (Rahman et al., 2006). This architecture contrasts with methylated flavonoids such as isorhamnetin, highlighting how structural variations dictate distinct mechanistic strategies in cancer therapy. Methylation favors stability and target selectivity, whereas polyhydroxylation drives redox and epigenetic modulation (Rana et al., 2025; Jeong et al., 2025). Figure 1 shows the sources and benefits of delphinidin. All figures were generated with BioGDP.com (Jiang et al., 2025). Delphinidin exhibits promising anti-cancer effects, most notably through its modulation of the tumor immune microenvironment, which points to its potential in combination with immunotherapy (Lin et al., 2017). This review offers a valuable resource for cancer research by summarizing the diverse anti-tumor mechanisms of delphinidin, thereby guiding future therapeutic exploration.

## 2 Structure, chemistry, and biosynthesis of delphinidin

### 2.1 Structure and chemistry

**FIGURE 2:**
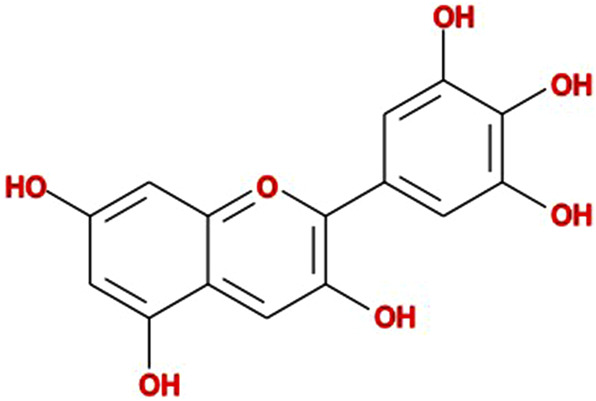
The chemical structure of delphinidin.

The chemical name of delphinidin is 3, 5, 7, 3′, 4′, 5′-hexahydroxyflavylium cation, with the molecular formula of C_15_H_11_O_7_^+^. The core chemical structure is a positively charged benzopyrylium ring (anthocyanidin nucleus) with three hydroxyl groups on ring B (3′, 4′, 5′-trihydroxy). This structural feature makes it an important blue pigment in nature, endowing it with powerful antioxidant abilities (Koss-Mikołajczyk and Bartoszek, 2023). In nature, delphinidin is rarely found as a free aglycone. Instead, it is typically bound to sugar molecules like glucose, galactose, or rhamnose through glycosidic bonds at its 3- or 5-position hydroxyl groups, forming more stable and water-soluble derivatives such as delphinidin-3-glucoside (Sharma et al., 2021; He and Giusti, 2010). The chemical structure of delphinidin is shown in Figure 2. The trihydroxylated B-ring is considered a key determinant for its redox and pro-apoptotic activities.

### 2.2 Biosynthesis

**FIGURE 3:**
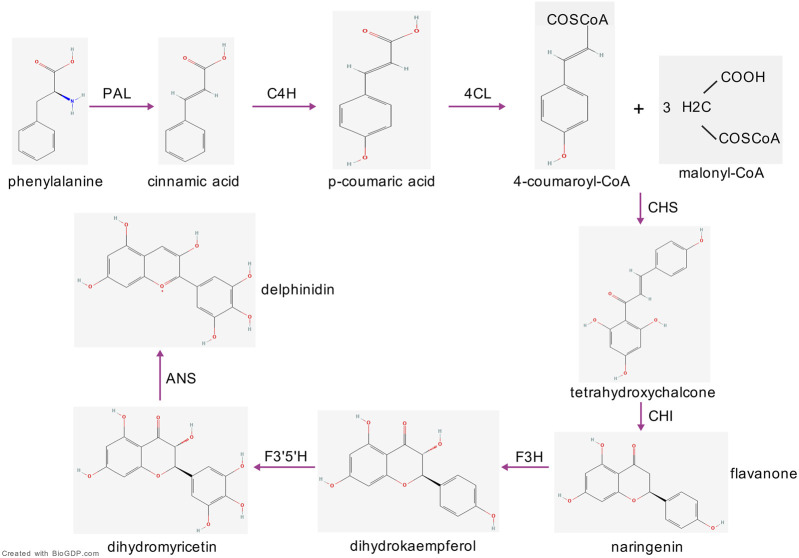
The biosynthesis of delphinidin. PAL, phenylalanine ammonia-lyase; C4H, cinnamate-4-hydroxylase; 4CL, 4-coumaroyl:CoA-ligase; CHS, chalcone synthase; CHI, chalcone isomerase; F3H, flavonoid 3-hydroxylase; F3′5′H, flavonoid 3′,5′-hydroxylase; ANS, anthocyanidin synthase.

The biosynthesis of delphinidin in plants begins with phenylalanine (Figure 3). Initially, phenylalanine ammonia-lyase catalyzes the deamination of phenylalanine to yield cinnamic acid, which is subsequently hydroxylated to produce 4-coumaric acid. This compound is then activated to form 4-coumaroyl-CoA. Subsequently, Chalcone synthase then catalyzes the condensation of 4-coumaroyl-CoA with malonyl-CoA to produce a chalcone, which is rapidly isomerized into a flavanone by chalcone isomerase (CHI) (Winkel-Shirley, 2001). This flavanone is subsequently hydroxylated by flavonoid 3-hydroxylase (F3H) to form a dihydroflavonol, such as dihydromyricetin. A crucial step involves the introduction of an additional hydroxyl group into the B-ring, a process catalyzed by flavonoid 3′,5′-hydroxylase. This modification results in a 3′,4′,5′-trihydroxylated structure, which serves as the direct precursor to delphinidin. This precursor is then oxidized by anthocyanidin synthase (ANS) to generate delphinidin (Winkel-Shirley, 2001). Finally, through modifications such as glycosylation and acylation, delphinidin is converted into stable derivatives (Zhao, 2015; Chen et al., 2019).

## 3 The anti-tumor mechanism of delphinidin

**FIGURE 4:**
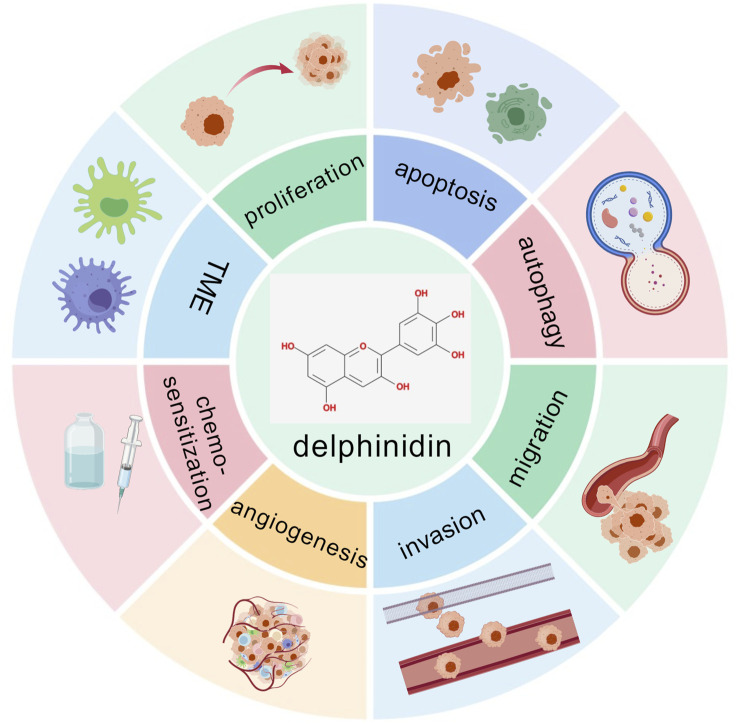
The role of delphinidin in cancer. TME: tumor immune microenvironment. The figure was created using BioGDP.com (Jiang et al., 2025).

**FIGURE 5:**
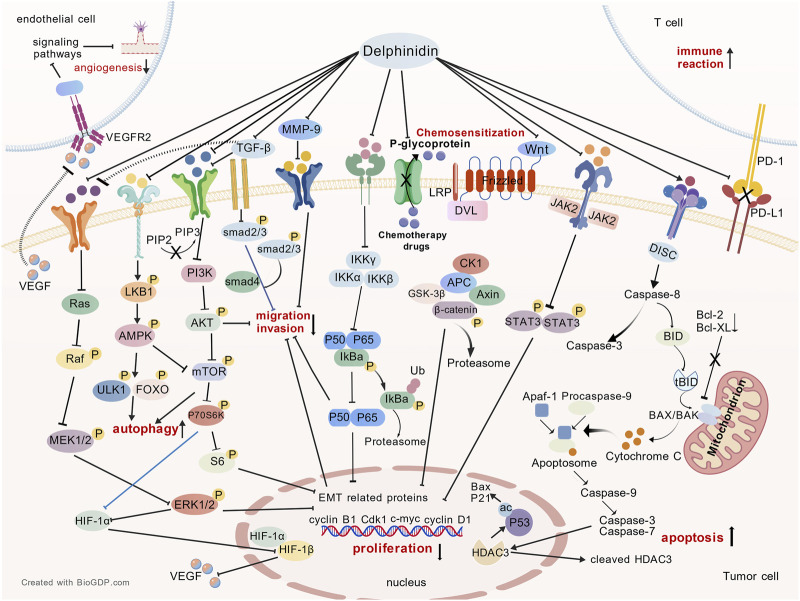
The specific molecular mechanisms of delphinidin against cancer. PD1, programmed cell death protein 1; PD-L1, programmed death-ligand 1; DISC, death-inducing signaling complex; BID, BH3-interacting domain death agonist; tBID, truncated BID; BAX, Bcl-2-associated X protein; BAK, Bcl-2 antagonist/killer, Apaf-1, apoptotic protease-activating factor 1; HDAC3, histone deacetylase 3; ac P53, acetylated p53; JAK2, Janus kinase 2; STAT-3, signal transducer and activator of transcription 3; CdK1, cyclin-dependent kinase 1; WNT, wingless-type MMTV integration site Family; LPR, low-density lipoprotein receptor-related protein; DVL, dishevelled; CK1, casein kinase 1; APC, adenomatous polyposis coli; Axin, axis inhibition protein; MMP-9, matrix metalloproteinase-9; EMT, epithelial-mesenchymal transition; Ub, Ubiquitination; TGF-β, transforming growth factor-beta; PI3K, phosphoinositide 3-kinase; AKT, protein kinase B; mTOR, mechanistic target of rapamycin; P70S6K, ribosomal protein S6 kinase beta-1; S6, ribosomal protein S6; LKB1, liver kinase B1; AMPK, AMP-activated protein kinase; ULK1, unc-51-like autophagy activating kinase 1; FOXO, forkhead box O; Ras, rat sarcoma virus; Raf, rapidly accelerated fibrosarcoma; MEK, MAPK/ERK Kinase; ERK, extracellular signal-regulated kinase; HIF, hypoxia-inducible factor; VEGF, vascular endothelial growth factor; VEGFR-2, vascular endothelial growth factor receptor-2. → promotion; ——I inhibition.

**FIGURE 6:**
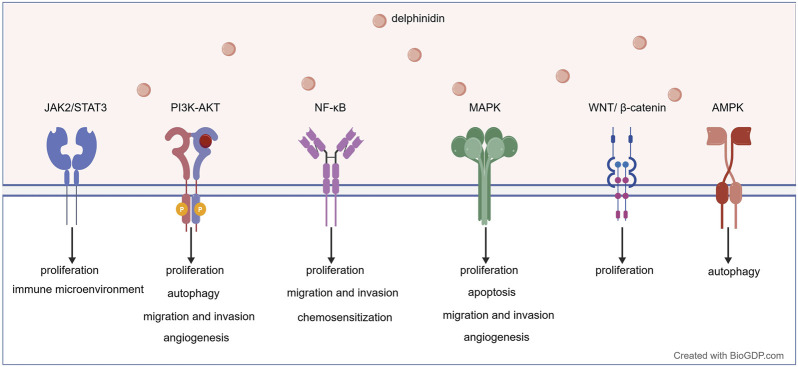
Multiple signaling pathways modulated by delphinidin to exert its anticancer effects. NF-κB: Nuclear Factor-kappa B.

Delphinidin exerts its multifaceted anticancer effects through coordinated modulation of key signaling networks that govern proliferation, apoptosis, autophagy, metastasis, angiogenesis, immune response and chemosensitization (Figure 4). As illustrated in Figures 5, 6, these pathways are not isolated but form an integrated signaling architecture.

### 3.1 Anti-proliferation

The antiproliferative activity of delphinidin was first reported in 2005, when anthocyanins were shown to significantly inhibit tumor cell growth (Zhang et al., 2005). Subsequent studies have revealed that delphinidin simultaneously modulates multiple parallel signaling pathways that converge on cell cycle regulation. Delphinidin targets the Nuclear Factor-kappa B (NF-κB) pathway by downregulating IKKα, thereby inhibiting IκBα phosphorylation and NF-κB p65 nuclear translocation, leading to G2/M cell cycle arrest (Yun et al., 2009; Wu et al., 2021, Gu et al., 2022). Independently, it promotes β-catenin phosphorylation, reducing nuclear translocation and downregulating Wingless/Integrated (Wnt) target genes including cyclin D1 and c-myc (Lee and Yun, 2016). In parallel, delphinidin inhibits additional oncogenic cascades, including Phosphoinositide 3-Kinase (PI3K)/protein kinase B (AKT), extracellular signal-regulated kinase (ERK) 1/2, Mitogen-Activated Protein Kinase (MAPK), and signal transducer and activator of transcription 3 (STAT-3) signaling, with studies confirming the functional relevance of these pathways in mediating its antiproliferative effects (Lim and Song, 2017; Zhang et al., 2021).

### 3.2 Induction of apoptosis

Among the six major anthocyanidins, only those with an ortho-dihydroxyphenyl structure on the B ring demonstrate pro-apoptotic activity. Delphinidin, the most potent among them, induces apoptosis by provoking oxidative stress. This is achieved through intracellular ROS generation, antioxidant depletion, lipid peroxidation, and single-strand DNA breaks, processes mediated by the c-Jun N-terminal kinase (JNK) signaling pathway (Hou et al., 2003; Zhang et al., 2021). Notably, delphinidin exhibits context-dependent dual roles in redox regulation. In normal cells, it acts as an antioxidant, maintaining redox homeostasis. In cancer cells, it shifts to a pro-oxidant, inducing ROS accumulation and mitochondrial-mediated apoptosis (Wang and Stoner, 2008). Overexpression of histone deacetylases (HDACs) is associated with various cancers. Novel epigenetic drugs targeting HDACs, particularly in the realm of natural products, have become a research hotspot (Karati et al., 2024). Delphinidin has been identified as a natural HDAC inhibitor, which leads to the acetylation and stabilization of p53 via caspase-mediated cleavage of HDAC3. Delphinidin effectively upregulates p53-positively regulated pro-apoptotic genes and downregulates the expression of various anti-apoptotic genes, thereby inducing cell apoptosis (Jeong et al., 2016). In addition, delphinidin treatment induces multiple hallmarks of apoptosis in cancer cells. These include Poly (ADP-ribose) polymerase (PARP) cleavage, activation of caspase-3 and caspase-9, upregulation of B-cell lymphoma 2 (Bcl-2)-associated X protein (Bax) and Bcl-2 antagonist/killer (Bak), and downregulation of Bcl-2, B-cell lymphoma-extra large (Bcl-xL), and myeloid cell leukemia 1 (Mcl-1) (Afaq et al., 2008).

### 3.3 Regulation of autophagy

Autophagy is a self-degradation process in which cytoplasmic cargo is delivered to lysosomes for degradation. Delphinidin modulates autophagy in cancer cells, with the functional outcome ranging from cytoprotective to cytotoxic depending on cancer type and cellular context. In HER2-positive breast cancer and osteosarcoma cells, delphinidin induces protective autophagy by targeting the mammalian target of rapamycin (mTOR) and AMP-activated protein kinase (AMPK) pathways, resulting in enhanced autophagosome formation and elevated levels of the autophagy hallmark microtubule-associated protein 1 light chain 3-II (LC3-II) (Chen et al., 2018; Lee et al., 2018). In hepatocellular carcinoma cells, delphinidin blocks autophagic flux, leading to significant accumulation of autophagosomes and a subsequent increase in apoptosis (Sun et al., 2023). As a glycoside flavonoid, purple sweet potato delphinidin-3-rutin (PSPD3R) triggers excessive autophagy that directly promotes apoptosis in glioblastoma cells, an effect mediated by the Akt/Creb/miR-20b-5p/Atg7 axis (Wang et al., 2022).

### 3.4 Inhibition of migration and invasion

Delphinidin exerts anti-metastatic effects by interfering with key signaling nodes that govern cell motility and invasion. It acts as a potent hyaluronidase inhibitor, directly impairing enzyme activity to reduce cancer cell motility (McGuire et al., 2024). Additionally, it interferes with epidermal growth factor (EGF)-induced epidermal growth factor receptor (EGFR) activation, thereby suppressing its downstream effectors Akt and ERK, which leads to inhibition of matrix metalloproteinase-2 (MMP-2) and reduced cell motility and invasion (Lim et al., 2019). It also attenuates brain-derived neurotrophic factor (BDNF)-promoted signaling by inhibiting Akt phosphorylation and subsequent NF-κB nuclear translocation (Lim et al., 2017). Through these direct actions on receptors and enzymes, delphinidin suppresses critical downstream cascades. Specifically, it blocks the MAPK pathway by reducing phosphorylation of ERK and p38, and decreases matrix metalloproteinase-9 (MMP-9) expression through coordinated inhibition of NF-κB signaling pathways (Kang et al., 2018; Im et al., 2014). Among various anthocyanins, delphinidin is the most effective epithelial mesenchymal transition (EMT) inhibitor. It significantly suppresses cancer cell migration by inhibiting the transforming growth factor-β (TGF-β) pathway, thereby altering the levels of mesenchymal markers such as fibronectin and Snail (Ouanouki et al., 2017). Delphinidin also reverses the EGF-driven EMT signature by upregulating E-cadherin and downregulating Vimentin and Snail (Lim et al., 2019). Furthermore, delphinidin restores the expression of downregulated MicroRNA-204-3p, which inhibits the αVβ3-integrin/FAK signaling pathway to further suppress EMT and metastasis (Huang et al., 2019). Collectively, these events result in reduced cancer cell migration and invasion.

### 3.5 Anti-angiogenesis

Inhibiting tumor angiogenesis is an effective strategy for delaying or blocking tumor growth (Yang et al., 2024). Among common anthocyanidins, delphinidin exhibits the strongest anti-angiogenic activity (Lamy et al., 2006; Barkallah et al., 2021). The anti-angiogenic activity of delphinidin is achieved primarily by inhibiting the vascular endothelial growth factor (VEGF)/ vascular endothelial growth factor receptor-2 (VEGFR-2) axis. It blocks VEGF-induced VEGFR-2 phosphorylation and the subsequent activation of ERK1/2 signaling. Consistent with this mechanism, delphinidin also suppresses angiogenesis *in vivo* in response to basic fibroblast growth factor (bFGF) (Lamy et al., 2006). Additionally, delphinidin suppresses hypoxia-inducible factor-1α (HIF-1α) expression by inhibiting the ERK and PI3K/Akt/mTOR pathways, which in turn reduces VEGF transcription and synthesis (Kim et al., 2017). Animal studies have shown that delphinidin inhibits EGF-induced neovascularization and downregulates the angiogenesis markers CD31 and VEGF in xenograft tumors (Kim et al., 2017; Pal et al., 2013). In human umbilical vein endothelial cells (HUVECs), delphinidin counteracts VEGF stimulation by not only suppressing cell migration but also reducing proliferation via G_0_/G_1_ phase cell cycle arrest (Favot et al., 2003).

### 3.6 Modulation of the immune microenvironment

The application of immunotherapy is one of the most exciting breakthroughs in the field of cancer treatment today. Delphinidin can enhance the immune response to tumors by promoting the activation and proliferation of anti-tumor immune cells. Delphinidin activates cytokine production by activating Ca (2+) release activated Ca (2+) (CRAC) channels and nuclear factor of activated T cells (NFAT), exerting an immune-stimulating effect on T cells (Jara et al., 2014). Programmed cell death protein 1 (PD-1)/programmed death-ligand 1 (PD-L1) is one of the most widely used immune checkpoint inhibitors in clinical applications (Ribas and Wolchok, 2018). Delphinidin-3-O-glucoside (D3G) and its metabolite, delphinidin, reduce the expression of the PD-L1 protein in tumor cells, activate immune responses within the tumor microenvironment, and induce apoptosis in cancer cells (Mazewski et al., 2019). Delphinidin may potentially restore T cell activity and regulate the tumor microenvironment by inhibiting the JAK2/STAT3 signaling pathway, downregulating the expression of PD-L1 in tumor cells and exosomes (Yu et al., 2024). In addition, delphinidin promotes the differentiation of regulatory T cells while inhibiting the function of memory T cells (Hyun et al., 2019). Given its ability to suppress PD-L1 expression, delphinidin may enhance the efficacy of immune checkpoint inhibitors, warranting further investigation into such combination strategies.

### 3.7 Chemotherapy sensitization

Chemoresistance is the major cause of poor prognosis in patients with advanced cancer (Al and Rabaan, 2023). Currently, most reversal drugs for multidrug resistance in tumors have serious side effects, hindering the pace of cancer treatment (Gottesman et al., 2023). Delphinidin has been reported to enhance the sensitivity of cancer cells to chemotherapy drugs. P-glycoprotein (P-gp) is an efflux transporter protein that pumps chemotherapy drugs out of cancer cells, leading to chemoresistance. During the process of malignant transformation in normal tissues, the expression of the P-gp transporter encoded by the multidrug resistance gene 1 (MDR1) gene increases (Lu et al., 2000). By inhibiting the expression of MDR1 and the pro-tumor cofactor DEAD-box Helicase 17 (DDX17), and promoting the activation of executors like cleaved caspase-3, delphinidin collectively enhances the apoptotic response in tumor cells (Sun et al., 2023). Computational docking indicates that delphinidin is a potential P-gp inhibitor, a finding that warrants further experimental validation (Diab et al., 2025). When combined with cisplatin, delphinidin significantly enhances the chemotherapeutic effect of cisplatin by inhibiting MDR1 expression (Sun et al., 2023). Methyl guanine methyl transferase (MGMT) is a DNA repair enzyme that mediates temozolomide resistance. Delphinidin-3-glucoside counteracts temozolomide resistance by inhibiting the key NF-κB/MGMT pathway (Carrillo-Beltrán et al., 2025). These findings align with the broader framework of flavonoid-drug synergy, wherein redox modulation, MDR transporter regulation, and apoptotic priming represent core mechanistic principles (Zou et al., 2024).

## 4 Cancer type-specific effects of delphinidin

**FIGURE 7:**
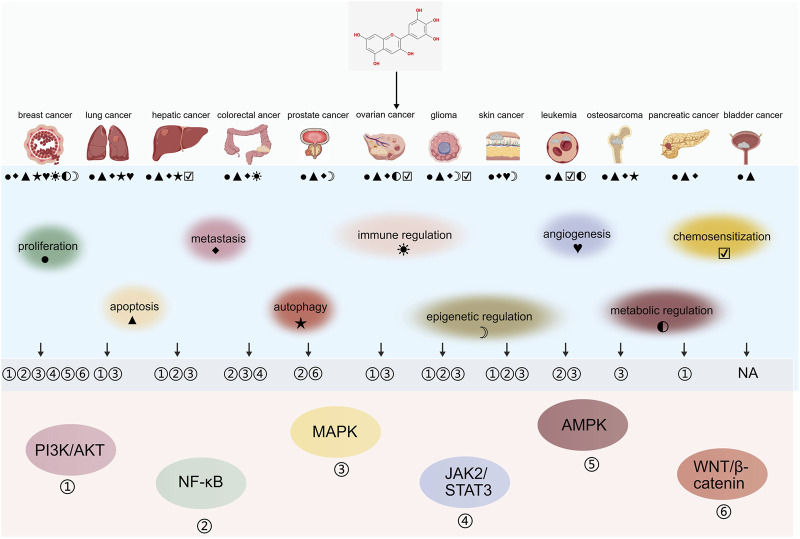
The anticancer effects of delphinidin across various tumor types and the involved signaling pathways. NA, not mentioned.

**FIGURE 8:**
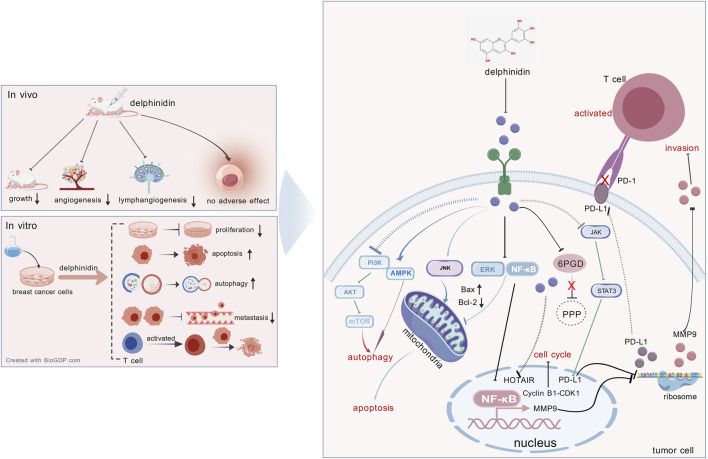
The anti-cancer role of delphinidin in breast cancer. 6PGD, 6-phosphogluconate dehydrogenase; PPP: pentose phosphate pathway.

Delphinidin exerts broad-spectrum anticancer effects across multiple tumor types via diverse mechanisms that differ by cancer type. The anticancer effects of delphinidin across various tumor types and the involved signaling pathways are summarized in Figure 7 and Table 1. Given the abundance of mechanistic studies on delphinidin in breast cancer, we have constructed a detailed figure (Figure 8) to integrate these complex signaling networks.

**TABLE 1:** The anticancer effects of delphinidin across different cancer types.

Type	Cell line/animal	Intervention	Mechanism	References
Breast cancer				
*In vitro*	AU-565, MCF-10A	Del (5,10,20,40 μM)/Del 3h + EGF (50 ng/mL)	cell viability↓,apoptosis↑, invasion↓, P-EGFR↓, PI3K↓, P-AKT↓,P-ERK1/2↓, P-JNK1/2↓, P-P38↓,BAX↑, Bcl-2↓, cleavage of PARP↑, caspase-3↑	Afaq et al. (2008)
*In vitro*	MCF-7,BT-474	Del (10,20,40,80,160 μM)	proliferation↓, apoptosis↑, CDK1↓,cyclin B1↓,P-c-Raf-1↓, P-MEK1/2↓, P-ERK1/2↓	Peng et al. (2022)
*In vitro*	HCC1806, MDA231, MDA468, SKBR3, MDA453, BT474, MCF7,MCF10A	Del (12.5–100 ug/mL)	proliferation↓, apoptosis↑, migration↓ P-HER2↓,P-AKT↓,P-ERK1/2↓	Ozbay and Nahta (2011)
*In vitro*	MDA-MB-453, BT-474	Del (10,20,40,80,160 μM)	cell viability↓,G (2)/M phase cell cycle arrest, apoptosis↑,CDK1↓,cyclin B1↓,p21^WAF1/Cip1^↑,BAX↑, Bcl-2↓,P-c-Raf-1↓, P-MEK1/2↓, P-ERK1/2↓, P-JNK↑,p-NF-κB/P65↓, p-IKKα/β↓, p-PKCα↓, IκBα↑, IKKα↑, IKKβ↑,PKCα↑,NF-κB/p65 nuclear translocation↓	Wu et al. (2021)
*In vitro*	MDA-MB-453, BT-474	Del (1.25–280 uM)	proliferation↓, apoptosis↑, autophagy↑ LC3-II/LC3-1↑,Atg5-Atg12↑,P-AKT↓,P-mTOR↓,P-eIF4e↓,P-p70s6k↓,P-LKB1↑,P-AMPK↑,P-FOXO3a↑,P-ULK1↑	Chen et al. (2018)
*In vitro*	MCF-7	Del (15,30,60,90 μM)	invasion↓,MMP-9↓,P-P38↓,P-JNK↓, nuclear translocation of p65↓,IκBα↑	Im et al. (2014)
*In vitro*	MDA-MB-453, BT-549,MCF-10A	Del (40,60,80,100 μM)	proliferation↓,migration↓,PD-L1↓,P-JAK2↓,P-STAT3↓ reinstates T-cell activity	Yu et al. (2024)
*In vivo* *In vitro*	MNU-induced female SD rat MDA-MB-231, MCF-7, DA-MB-453	100 mg/kg/d/rat 40 uM	proliferation↓, no adverse effect, HOTAIR↓ cell viability↓,migration↓, inavsion↓ HOTAIR↓, miR-34a↑, β-catenin↓, P-GSK-3β↓, c-Myc↓, cyclin-D1↓, MMP-7↓	Han et al. (2019)
*In vivo*	Wistar-Furth rat	1.18 × 10^−5^mol of Del, daily	angiogenesis↓,lymphangiogenesis↓	Thiele et al. (2013)
*In vitro* *In vivo*	MCF-7 Swiss albino male and female mice	Pomegranate peel extract (one of potent phytochemicals is Del), 2000 mg/kg/mouse 0.3–200 ug/mL	non-toxicity cell viability↓	Riaz et al. (2025)
Lung cancer				
*In vitro* *In vivo*	A549,H1299 Female athymic nude (nu/nu) mice	Del (25, 50,75,100 µM) Del 1.5mg/mouse on alternate days	cell viability↓ tumor growth↓	Kausar et al. (2012)
*In vitro*	A549	Del (0–50 uM) Del+γ ray irradiation	cell viability↓,apoptosis↑ autophagy↑,LC3-II/LC3-I↑,Atg5↑,Atg12↑,P-PI3K↓,P-AKT↓,P-mTOR↓ p53↑,DRAM↑,P-ERK↓, P-JNK↑	Kang et al. (2020)
*In vitro*	A549	Del (0–50 uM) Del + Oroxylin A	cell viability↓ apoptosis↑, migration↓ ROS↑,G (2)/M phase cell cycle arrest,P-STAT3↓,cyclin D1↓ BAX↑,Bcl-2↓,P-FAK↓,MMP-2↓	Wan et al. (2024)
*In vitro* *In vivo*	NCI-H441,SK-MES-1,A549 female athymic (nu/nu) nude mice	Del (5,10,20,40,60 μM) 1 mg,2mg/animal, 3 times/week	cell viability↓, apoptosis↑, P-EGFR↓ P-VEGFR2↓,PI3K↓,P-AKT↓,P-ERK↓,P-JNK↓, P-P38↓,cyclin D1↓, PCNA↓ Bcl2↓,Bcl-xL↓,Mcl-1↓, BAX↑,BAK↑ tumor growth↓, Ki67↓, PCNA↓, active caspase-3↑,CD31↓, VEGF↓	Pal et al. (2013)
*In vitro* *In vivo*	A549 C57BL/6N mice	Del (10, 20, 40,80 µM) Del + CoCl2 (200 µM) Del + EGF (20 ng/mL) Del (0, 20, 40,80 µM) +EGF (0.200 ng/mL)	angiogenesis↓,HIF-1α↓,VEGF↓, P-ERK↓,PI3K/Akt/mTOR/p70S6K phosphorylation ↓ tumor angiogenesis↓	Kim et al. (2017)
Hepatic cancer				
*In vitro*	HepG2	Del (50, 100, 150,200 µM)	cell viability↓, apoptosis↑,LDH leakage↑,DNA fragmentation↑, caspase-3 activation↑, c-Jun↑, p-JNK↑,intracellular ROS↑, Bax↑, Bcl-2↓	Yeh and Yen (2005)
*In vitro*	HepG2, HuH-7	Del (10, 20, 30, 40,50 μg/mL)	cell viability↓, apoptosis↑, autophagic flux blockage↑,autophagosomes↑, Lc3BII/I↑, P62↑, cleaved caspase3↑,p-JNK↑,p-p38↑,p-p65↓ chemotherapy efficiency↑,MDR1↓,DDX17↓	Sun et al. (2023)
*In vitro*	SMMC7721	Del (80, 100, 150 µM)	cellular vacuolization↑, LC3 lipidation↑, growth retardation	Feng et al. (2010)
*In vitro*	Huh7, PLC/PRF/5	Del (30,40,80,100 µM) Del + EGF (100 ng/mL)	cell viability↓,EGF-induced EMT↓,E-cadherin↑,vimentin↓,Snail↓ EGF-induced migration and invasion↓, MMP-2↓,p-EGFR↓, p-AKT↓, p-ERK↓	Lim et al. (2019)
Colorectal Cancer				
*In vitro*	HCT116	Del (80,100,120 µM)	cell viability↓,intracellular ROS↑,MMP↓,DNA damage↑,apoptosis↑,p-STAT3↓,P-JAK2↓, p-p38↓, p-ERK1/2↓,Bax↑,Bad↑, caspase- 3↑, caspase-8↑,caspase- 9↑, cytochrome C↑,Bcl-2↓,Bcl-XL↓	Zhang et al. (2021)
*In vitro*	HT29	Del (0.1,1,10,20 30,50,100 µM) EGF (100 ng/L)	P-EGFR↓, p-ERK1/2↓	Fridrich et al. (2008)
*In vitro*	HCT116	Del (30, 60, 120, 180, 240 µM)	cell viability↓,apoptosis↑, cleaved PARP↑, procaspase-3↓, procaspase-8↓, procaspase-9↓, Bcl-2↓, Bax↑, G2/M phase cell cycle arrest, cyclin B1↓, cdc2↓, p53↑, p21^WAF1/Cip1^↑, and NF-κB activation↓	Yun et al. (2009)
*In vitro* *In vivo*	DLD-1, SW480, SW620 Male Balb/c nude mice	Del (20, 40, 60, 80, 100 µM) DLD-1 implantation + Del (100 μM)	colony formation and adhesion↓ migration↓, invasion↓, EMT↓,Snail↓, Slug↓, Twist↓, β-catenin↓, MMP-2↓, E-cadherin↑,miR-204-3p↑,integrin αV/β3↓, integrin/FAK signaling cascade↓ metastasis↓ liver weight (−)	Huang et al. (2019)
*In vitro*	HT29	Del 25 μg/mL + H_2_O_2_ (50 µM), 24 h	H_2_O_2_-induced PGK-1 expression↓	Jang et al. (2008)
*In vitro*	HCT116,HT29,PBMCs co-cultured with HCT-116 and HT-29	D3G and its metabolites delphinidin chloride (50,100,200,400,600 μg/mL),24h	apoptosis↑,VEGF↓, PD-L1 ↓, PD-1 ↓, binding of PD-L1 to PD-1 ↓	Mazewski et al. (2019)
prostate cancer				
*In vitro* *In vivo*	PC3 athymic (nu/nu)male nude mice	Del (15, 30, 60, 90, 120,180 µM) 2 mg/animal in 100 µL of 1:10 ratio of DMSO and normal saline), thrice a week	cell viability↓,apoptosis↑,PARP cleavage↑,Bax/Bcl2 ratio↑, caspase3↑,caspase9↑,G2/M phase cell cycle arrest, p27/KIP1↑, p21/WAF1↑,cyclin D1↓, cyclin A↓, cdk1↓,cdk2↓, activity of NF-κB↓,p- IκBα↓,p-IKKγ↓ tumorigenicity↓,Bax↑,Bcl-2↓, cyclin D1↓,NF-κB↓,Ki67↓,PCNA↓	Hafeez et al. (2008)
*In vitro*	PC3	Del (15, 30, 60, 120, 180, 240 µM)	cell viability↓,β-catenin↓,Axin2↓, cyclin D1↓, c-myc↓,TCF1↓,LEF1↓ p-β-catenin↑,β-catenin destruction complex↑,E-cadherin↑	Lee and Yun (2016)
*In vitro*	22Rv1	Del (30, 60, 90,120 µM)	cell viability↓,apoptosis↑,PARP cleavage↑,Bax/Bcl2 ratio↑, caspase3↑,caspase9↑,G2/M phase cell cycle arrest, NF-κB signaling↓,NFκB Mediated Transcription Activation↓,p- IκBα↓,p-IKKγ↓	Bin Hafeez et al. (2008)
*In vitro*	RM1	Del (15,30 µM)	proliferation↓,migration↓, invasion↓	McGuire et al. (2024)
*In vitro*	LNCaP	Del (50,100,150 µM)	apoptosis↑, caspase activity↑, HDAC3↓, p53 acetylation↑,Bax↑, Puma↑, Noxa↑ p21↑	Jeong et al. (2016)
*In vitro*	LNCaP	Del (30,60,90 µM) TRAIL (0, 25,50, 100, 150 ng/mL)	proliferation↓, cleaved PARP↑, caspase-8↑, caspase-9↑, cleaved caspase-3↑, caspase-7↑, DR5↑, p21↑ Bax↑, Bcl-2↓, XIAP↓, cIAP-2 ↓, Mcl-1↓, survivin↓, HDAC3↓,p53 acetylation↑,p53↑	Ko et al. (2015)
Ovarian cancer				
*In vitro*	SKOV3	Del (0.1,1,10 µM)	cell viability↓,apoptosis↑, p-Akt↓, p-p70S6K↓, pS6↓, p-ERK1/2↓, p-P38↓,G0/G1 and G2/M phases cell cycle arrest, chemotherapeutic activity of paclitaxel ↑	Lim and Song (2017)
*In vitro*	ES2	Del (0.1,1,10,50,100 µM)	cell viability↓, migration↓, apoptosis↑, p-Akt↓,p-p70S6K↓, p-ERK1/2↓ p-JNK↓,chemotherapeutic activity equivalent to cisplatin or paclitaxel	Lim et al. (2016)
*In vitro*	SKOV3	Del (5,10,50,75,100,200 µM) BDNF (100 nM)	cell viability↓, migration↓, MMP-2↓ MMP-9↓, p-Akt↓, nucleus translocation of NF- κB↓	Lim et al. (2017)
*In vitro*	SKOV3, PEO1	Del (10–100 µM) 3-BP(5–25 µM for PEO1, 10–50 µM for SKOV3)	cell viability↓, ATP level↓,necrosis↑ DHE-detectable ROS↑, DCFDA-reactive ROS↑(PEO1), mitochondrial potential↓(PEO1), mitochondrial potential↑(SKOV3), mitochondrial mass↑(PEO1) mitochondrial mass↓(SKOV3) migration↓,apoptosis↑(SKOV3)	Pieńkowska et al. (2021)
Glioma				
*In vitro*	U87-MG	glycosylated Del (15, 30, 60, 80, 100, 120, 180, 240 µM)	cell viability↓,NF-κB activity↓, STING↓,SHARPIN↓,MGMT↓,the sensitivity of U87-MG to TMZ↑	Carrillo-Beltránet al. (2025)
*In vitro*	U87	Del 25 µM Del (25,50, 75,100 µM)	migration↓, invasion↓,uPAR↓, LPR↓,uPA↑,PAI-1↓,uPA-dependent conversion of plasminogen to plasmin↓	Lamy et al. (2007)
*In vitro*	U87-MG	Del (35,50 µM) TGF-β (10 ng/mL)	migration↓ cell viability↓ TGFβ/p-Smad2↓, TGFβ/p-ERK↓, fibronectin↓, Snail↓	Ouanouki et al. (2017)
*In vitro*	U87-MG,LN18	Del (10, 25, 50 µM) AzaC (5, 10, 20 μM)	cell viability↓,invasion↓,apoptosis↑, miR-137↑, p-Akt↓, NF-κB↓, VEGF↓, b-FGF↓, EGFR↓, MMP-9↓, MMP-2↓, angiogenic network formation↓ caspase-8↑, truncated Bid↑, Bax↑, caspase-3↑, caspase-9↑, Bcl-2↓,ICAD fragment↑	Chakrabarti and Ray (2015)
Skin cancer				
*In vitro* *In vivo*	JB6 P+ Female ICR mice	Del (5, 10,20 μM) UVB,0.5 kJ/m^2^ Del (0, 40,200 nmol) UVB,5 kJ/m^2^	COX-2↓,PGE2 production↓, transactivation of AP-1 and NF-κB↓, p-JNK1/2↓,p-c-Jun↓,p-p38↓,p-Akt↓ p-ATF2↓,p-ERK1/2↓,p-p90RSK↓,p-p70S6K↓,MAPKK4 and PI-3K activity↓ COX-2↓,MAPKK4 and PI-3K activity↓	Kwon et al. (2009)
*In vitro*	JB6 P+	Del (10, 20, 40 µM) TNF-α, 5 ng/mL	COX-2↓, AP-1 and NF-kB transcription activities↓, p-JNK↓, p-p38 MAP kinase↓,p-Akt↓, p-p90RSK↓, p-MSK1↓, p-ERK↓, Fyn kinase activity↓	Hwang et al. (2009)
*In vitro*	JB6 P+	Del (5,10, 20, 40 µM) TPA,10 ng/mL	neoplastic transformation ↓,COX-2 ↓, PGE2↓, AP-1 and NF-kB transcription activities↓, c-fos promoter activity↓, p-MEK↓,p-ERK↓, p-90RSK↓, p-MSK↓, Raf1 and MEK1 activities↓	Kang et al. (2008)
*In vitro*	JB6 P+	Del (10, 20, 40, 60, 80, 100 μM) TPA,10 ng/mL	cell viability↓,Nrf2↑, HO-1↑, Nqo1↑, SOD1↑, CpG methylation↓ DNMTs↓, HDACs↓	Kuo et al. (2019)
*In vitro* *In vivo*	B16-F10 HUVECs C57BL/6N mice	Del 10ug/mL VEGF 10 ng/mL 10 mg delphinidin/kg, twice at 7-day intervals (D16 and D23)	basal and VEGFR2-mediated endothelial cell proliferation↓ Tumor weight↓	Keravis et al. (2015)
*In vitro* *In vivo*	B16-F10 wild type C57BL/6 mice	Del (15,30 µM) Del,50 mg/kg,three times a week	proliferation↓,migration↓,invasion metastasis↓	McGuire et al. (2024)
*In vitro* *In vivo*	B16 C57BL/6J male mice	Del (1.10 µM) Del, 20 mg/kg of body weight	proliferation↓,cyclin D1↓ increasing let-7b expression through Fam222B let-7b↑	Murata et al. (2025)
Leukemia				
*In vitro*	HL-60	Del 20 µM	apoptosis↑	Feng et al. (2010)
*In vitro*	HL-60	Del 200 µM	apoptosis↑	Katsube et al. (2003)
*In vitro*	HL-60	Del (10,30,100 µM)	cell viability↓,apoptosis↑ inhibition of Glyoxalase I	Takasawa et al. (2010)
*In vitro*	HL-60	Del (20,40,60,80,100,120 µM)	apoptosis↑,c-Jun↑, p-JNK↑, caspase-3↑, intracellular hydrogen peroxide↑	Hou et al. (2003)
*In vitro*	HL-60	Del (5,20,50 µM) Del (8 µM), As(III) (5 µM)	cell viability↓,apoptosis↑, cleaved forms of caspase-8,caspase-9 and caspase-3↑, Bid↓,loss of mitochondrial membrane potential, intracellular GSH↓, NF-κB-binding activity↓	Yoshino et al. (2018)
*In vitro*	NB4	Del (0.3, 1, 3, 10, 20, 30 µM) Del (8 µM), As(III) (2 µM)	cell viability↓,apoptosis↑,cleaved forms of caspase-8,caspase-9 and caspase-3↑, Bid↓, loss of mitochondrial membrane potential, enhanced cytotoxic effect	Yuan et al. (2015)
osteosarcoma				
*In vitro*	HOS, U2OS	Del (10, 25, 50, 75, 100 µM)	cell viability↓,apoptosis↑, migration↓ invasion↓,EMT↓,Bcl-2↓,Bak↑, cleavage caspase-3, cleaved PARP↑, E-cadherin↑, N-cadherin↓, Snail↓, Slug↓, P-ERK↓, p- P38↓	Kang et al. (2018)
*In vitro*	U2OS	Del (10, 50, 100, 200 μg/mL)	cell viability↓, ROS↑, LC3-II↑, autophagosome formation↑, p62↓	Lee et al. (2018)
Pancreatic Cancer				
*In vitro* *In vivo*	BxPc-3, PANC-1 Male BALB/c nude mice	Del (50,100,150,200 μg/mL) Del, 50μM and 100 μM	cell viability↓,apoptosis↑,G0/G1 phase cell cycle arrest, invasion↓,p53↑ p-AKT↓,p-PI3K↓ metastasis↓	Wang et al. (2024)
Bladder cancer				
*In vitro*	T24	Del (10, 20, 30, 40, 50, and 60 μg/mL)	cell viability↓,ROS↑,sub-G1 proportion of cells↑, apoptosis↑	Wang et al. (2021)

DEL, delphinidin; EGF, epidermal growth factor; P-EGFR, phosphorylation of epidermal growth factor receptor; DRAM, damage-regulator autophagy modulator; PGK, phosphoglycerate kinase; PBMCs, peripheral blood mononuclear cells; DMSO, dimethyl sulfoxide; DR5, death receptor 5; XIAP, X-linked inhibitor of apoptosis protein; DHE, dihydroethidium; DCFDA, 2′,7′-dichlorofluorescein; MGMT, methyl guanine methyl transferase; TMZ, temozolomide; uPAR, urokinasetype plasminogen activator receptor; uPA, urokinase-type plasminogen activator; PAI-1, plasminogen activator inhibitor-1; LRP, lipoprotein receptor-related protein; NU, 1-methyl-1-nitrosourea; SD, Sprague-Dawley; UVB, Ultraviolet B; ICR, institute of cancer research; COX-2, Cyclooxygenase-2; PGE2, prostaglandin E 2; Nrf2, nuclear factor E2-related factor 2; HO-1, Heme oxygenase-1; NQO1, NAD(P)H/quinone oxidoreductase 1; SOD, superoxide dismutase; TPA, 12-O-tetradecanoylphorbol-13-acetate; DNMTs, DNA, methyltransferases; TMZ, temozolomide; AzaC, 5-Aza-2-deoxycytidine; ICAD, inhibitor of caspase-activated DNase; ATF2, activating transcription fractor 2; HDACs, histone deacetylases; FAM222B, Family With Sequence Similarity 222 Member B; ROS, reactive oxygen species.

Delphinidin consistently suppresses cancer cell proliferation by targeting the PI3K/Akt/mTOR and MAPK/ERK signaling axes across multiple cancer types. Inhibition of PI3K/AKT and ERK1/2 signaling, accompanied by reduced proliferative capacity, is observed in ovarian, pancreatic and breast cancer cells (Lim and Song, 2017; Afaq et al., 2008; Ozbay and Nahta, 2011; Lim et al., 2016; Wang et al., 2024). In prostate cancer, delphinidin suppresses proliferation by inhibiting NF-κB activation (Gu et al., 2022). In colorectal cancer, delphinidin inhibits cell proliferation by suppressing STAT-3 and MAPK (p38, ERK1/2) phosphorylatio (Zhang et al., 2021). In non-small cell lung cancer, delphinidin reduces cell proliferation and induces apoptosis through inhibition of EGFR/VEGFR2 pathways (Pal et al., 2013). Delphinidin treatment results in cell cycle arrest, inducing G0/G1 arrest in human umbilical vein endothelial cells and G2/M arrest in HER2-positive breast, lung, colorectal, prostate, and ovarian cancer cells, thereby suppressing tumor growth (Yun et al., 2009; Wu et al., 2021; Lim and Song, 2017; Favot et al., 2003; Wan et al., 2024; Bin Hafeez et al., 2008).

Beyond proliferative control, delphinidin actively tips the balance toward apoptosis through multiple mechanisms. In breast cancer, it inhibits ERK and NF-κB while activating JNK to promote mitochondrial apoptosis (Wu et al., 2021). It activates JNK-mediated apoptotic signaling and modulates Bcl-2 family proteins in leukemia and liver cancer (Hou et al., 2003; Yeh and Yen, 2005; Alhosin et al., 2015). Caspase-dependent apoptosis is also observed in leukemia, prostate, glioma and osteosarcoma cells following delphinidin treatment (Hou et al., 2003; Kang et al., 2018; Yuan et al., 2015; Jeong, et al., 2016). Autophagy plays a context-dependent role in response to delphinidin. It induces protective autophagy via the mTOR/AMPK pathway in breast cancer, but disrupts autophagic flux to promote apoptosis by suppressing MDR1/DDX17 in liver cancer. (Chen et al., 2018; Sun et al., 2023).

Delphinidin potently inhibits tumor cell migration and invasion through multiple context-dependent mechanisms. It inhibits NF-κB-dependent MMP-9 expression in breast cancer, reducing invasive capacity (Im et al., 2014). In colorectal cancer, it blocks integrin/FAK signaling to inhibit lung metastasis (Huang et al., 2019). In ovarian cancer, it attenuates BDNF-induced cell motility (Lim et al., 2017). Delphinidin also counteracts EMT by targeting EGFR/AKT/ERK in liver cancer, MAPK pathways in osteosarcoma, and TGF-β signaling in glioma (Lim et al., 2019; Kang et al., 2018; Ouanouki et al., 2017). In parallel, anti-angiogenic effects are among the most conserved activities of delphinidin. In A549 lung cancer cells, delphinidin inhibits angiogenesis through suppression of HIF-1α and VEGF expression (Kim et al., 2017). It also blocks the VEGF/VEGFR-2 axis and downstream ERK1/2 activation, and inhibits bFGF-induced angiogenesis *in vivo* (Lamy et al., 2006). Furthermore, delphinidin reduces CD31 and VEGF expression in xenograft tumors and inhibits EGF-induced neovascularization (Kim et al., 2017; Pal et al., 2013). These combined anti-metastatic and anti-angiogenic activities position delphinidin as a potent inhibitor of tumor dissemination.

In addition to these canonical pathways, delphinidin functions as an epigenetic modulator in multiple cancers. In prostate cancer, it inhibits HDAC3 activity and promotes p53 acetylation, inducing p53-mediated apoptosis (Jeong et al., 2016). In skin cancer, it demethylates the nuclear factor erythroid 2-related factor 2 (Nrf2) promoter to activate antioxidant responses (Kuo et al., 2019; Thoppil et al., 2012). It also modulates non-coding RNAs by targeting the HOTAIR/miR-34a axis in breast cancer, upregulating let-7b to suppress cyclin D1 in melanoma, and synergizing with miR-137 upregulation in glioma (Zhao et al., 2023; Han et al., 2019; Murata et al., 2025; Chakrabarti and Ray, 2015).

Emerging evidence further highlights delphinidin’s role as an immune modulator and metabolic regulator. In breast cancer, it enhances T-cell killing through the JAK2/STAT3/PD-L1 axis (Yu et al., 2024). In colorectal cancer, delphinidin-3-O-glucoside downregulates PD-L1 to stimulate anti-tumor immune responses (Mazewski et al., 2019). Metabolically, delphinidin inhibits 6-phosphogluconate dehydrogenase (6PGD), a pentose phosphate pathway enzyme overexpressed in breast cancer (Riaz et al., 2025). It sensitizes leukemia cells to arsenite via glutathione depletion and synergizes with glycolytic inhibitor 3-bromopyruvate in ovarian cancer (Yuan et al., 2015; Yoshino et al., 2018; Pieńkowska et al., 2021).

Finally, delphinidin enhances the efficacy of multiple conventional therapeutics. It exhibits synergistic effects with cisplatin in liver cancer, oroxylin A in lung cancer, temozolomide and 5-aza-2′-deoxycytidine in glioma, and arsenite in leukemia (Sun et al., 2023; Carrillo-Beltrán et al., 2025; Wan et al., 2024; Chakrabarti and Ray, 2015; Yoshino et al., 2018). Notably, it retains efficacy in paclitaxel-resistant ovarian cancer cells, suggesting potential for overcoming chemoresistance (Lim and Song, 2017).

## 5 Limitations, challenges and prospects of delphinidin

Anthocyanins, including delphinidin, exhibit very low oral bioavailability due to their poor absorption in the gastrointestinal tract and extensive intestinal and hepatic first-pass metabolism, resulting in limited plasma concentration (He and Giusti, 2010). Additionally, delphinidin is prone to degradation under high pH conditions, leading to reduced bioactivity (Fleschhut et al., 2006). Therefore, delphinidin faces a fundamental challenge posed by the substantial gap between its effective *in vitro* concentrations and actual *in vivo* exposure levels. Human tracer studies show that plasma concentrations of parent anthocyanins are typically in the low nanomolar range and short-lived (Ekundayo et al., 2025). For instance, a study reported that after human volunteers ingested a mixture of black currant anthocyanins (BCA) at a dose of 6.24 μmol/kg body weight, the plasma Cmax of delphinidin-3-rutinoside was only 73.4 ± 35.0 nmol/L (Matsumoto et al., 2001). However, the effective concentrations of delphinidin in the *in vitro* studies reviewed here are mostly in the µM range. This is hundreds to thousands of times higher than actual human exposure levels. This “translational gap” is a core reason for the failure of natural product research to achieve clinical success.

Several strategies have been proposed to address these limitations. A range of delivery systems, such as nanoemulsions, nanoliposomes, microcapsules, and hydrogels, can effectively enhance the solubility and stability of delphinidin, thereby improving its bioavailability (Cheng et al., 2023). Furthermore, structural modifications such as glycosylation, acylation, pyranization have been shown to enhance its bioavailability and efficacy (Xue et al., 2024). A study has utilized the properties of small extracellular vesicles (sEVs) to improve the stability and efficacy of delphinidin (Barkallah et al., 2021). Delphinidin loaded into sEVs has been shown to act at different steps of angiogenesis, suggesting that sEVs may be a promising method for delivering delphinidin to target angiogenesis-related diseases including cancer. A formulation composed of bilberry anthocyanins, chitosan, and pectin was shown to modulate gut microbiota through “biotic stimulation,” increase beneficial metabolites such as butyrate, and promote antitumor T-cell infiltration. This significantly enhanced the efficacy of PD-L1 immune checkpoint inhibitor against colorectal cancer (Liu et al., 2020).

Although delphinidin has shown promising anticancer prospects in preclinical studies, the trial data supporting its clinical translation remains insufficient, which remains a major obstacle to its development. The maqui berry (Aristotelia chilensis) is recognized as the most abundant natural source of delphinidin, from which delphinidin is extracted and standardized to a concentration of 25% (Watson and Schönlau, 2015). In a double-blind randomized controlled clinical trial, the intervention involving Delphinol® was shown to improve oxidative stress status in healthy adults, overweight individuals, and adult smokers (Davinelli et al., 2015). In a separate trial with healthy Japanese women, daily intake was shown to support facial skin health (Norihito et al., 2020). Moreover, Delphinol® significantly and dose-dependently reduced fasting blood glucose levels and insulin concentrations in prediabetic individuals (Alvarado et al., 2016). Future clinical trials investigating the anticancer properties of delphinidin are necessary to draw reliable conclusions and facilitate clinical translation.

## 6 Conclusion

As a natural plant compound, delphinidin has gradually attracted attention in tumor therapy in recent years. Current research indicates that delphinidin can inhibit tumor growth and progression through multiple mechanisms. Delphinidin may be a potential natural chemotherapy drug or chemotherapy sensitizer. Combining delphinidin with conventional chemotherapy drugs is expected to augment treatment response and mitigate side effects. The anti-angiogenic and immune microenvironment regulatory effects of delphinidin suggest that it may demonstrate promising application prospects as an adjuvant drug in anti-tumor therapy.

Although delphinidin shows promising prospects in anti-tumor therapy, it still faces some challenges. The underlying mechanisms of delphinidin across different cancer types remain incompletely understood. Future research should focus on delineating its mode of action in various cancers to facilitate clinical translation. Additionally, it is necessary to further explore the optimal dosage, administration route, and the best combination of delphinidin with other drugs. Due to the limited number of clinical trials related to delphinidin’s anti-cancer properties, its safety and long-term effects in clinical applications cannot be verified, thus requiring large-scale clinical trials for research.

In conclusion, delphinidin demonstrates anticancer effects through various mechanisms, highlighting its significant potential in oncology research. Therefore, future efforts should prioritize the transition from preclinical studies to clinical trials, aimed at evaluating its safety and efficacy, ultimately providing innovative therapeutic strategies for cancer patients.

## References

[B1] Afaq F. Zaman N. Khan N. Syed D. N. Sarfaraz S. Zaid M. A. (2008). Inhibition of epidermal growth factor receptor signaling pathway by delphinidin, an anthocyanidin in pigmented fruits and vegetables. Int. J. Cancer. 123, 1508–1515. 10.1002/ijc.23675 18623129

[B3] Al S. H. A. Rabaan A. A. (2023). Cellular resistance mechanisms in cancer and the new approaches to overcome resistance mechanisms chemotherapy. Saudi Med. J. 44, 329–344. 10.15537/smj.2023.44.4.20220600 37062547 PMC10153614

[B4] Alhosin M. León-González A. J. Dandache I. Lelay A. Rashid S. K. Kevers C. (2015). Bilberry extract (antho 50) selectively induces redox-sensitive caspase 3-related apoptosis in chronic lymphocytic leukemia cells by targeting the Bcl-2/Bad pathway. Sci. Rep. 5, 8996. 10.1038/srep08996 25757575 PMC4355738

[B5] Alvarado J. L. Leschot A. Olivera-Nappa Á. Salgado A. M. Rioseco H. Lyon C. (2016). Delphinidin-rich maqui berry extract (delphinol®) lowers fasting and postprandial glycemia and insulinemia in prediabetic individuals during oral glucose tolerance tests. Biomed. Res. Int. 2016, 9070537. 10.1155/2016/9070537 28025651 PMC5153493

[B6] Barkallah M. Nzoughet-Kouassi J. Simard G. Thoulouze L. Marze S. Ropers M. H. (2021). Enhancement of the anti-angiogenic effects of delphinidin when encapsulated within small extracellular vesicles. Nutrients 13, 4378. 10.3390/nu13124378 34959929 PMC8703615

[B7] Bin Hafeez B. Asim M. Siddiqui I. A. Adhami V. M. Murtaza I. Mukhtar H. (2008). Delphinidin, a dietary anthocyanidin in pigmented fruits and vegetables: a new weapon to blunt prostate cancer growth. Cell. Cycle 7, 3320–3326. 10.4161/cc.7.21.6969 18948740 PMC2989799

[B8] Bray F. Laversanne M. Sung H. Ferlay J. Siegel R. L. Soerjomataram I. (2024). Global cancer statistics 2022: GLOBOCAN estimates of incidence and mortality worldwide for 36 cancers in 185 countries. Ca. Cancer. J. Clin. 74, 229–263. 10.3322/caac.21834 38572751

[B9] Carrillo-Beltrán D. Nahuelpan Y. Cuevas C. Fabres K. Silva P. Zubieta J. (2025). Glycosylated delphinidins decrease chemoresistance to temozolomide by regulating NF-κB/MGMT signaling in glioblastoma. Cells 14, 179. 10.3390/cells14030179 39936970 PMC11816850

[B10] Chakrabarti M. Ray S. K. (2015). Direct transfection of miR-137 mimics is more effective than DNA demethylation of miR-137 promoter to augment anti-tumor mechanisms of delphinidin in human glioblastoma U87MG and LN18 cells. Gene 573, 141–152. 10.1016/j.gene.2015.07.034 26187071

[B11] Chen J. Zhu Y. Zhang W. Peng X. Zhou J. Li F. (2018). Delphinidin induced protective autophagy *via* mTOR pathway suppression and AMPK pathway activation in HER-2 positive breast cancer cells. Bmc. Cancer. 18, 342. 10.1186/s12885-018-4231-y 29587684 PMC5870693

[B12] Chen Z. Zhang R. Shi W. Li L. Liu H. Liu Z. (2019). The multifunctional benefits of naturally occurring delphinidin and its glycosides. J. Agric. Food. Chem. 67, 11288–11306. 10.1021/acs.jafc.9b05079 31557009

[B13] Cheng Y. Liu J. Li L. Ren J. Lu J. Luo F. (2023). Advances in embedding techniques of anthocyanins: improving stability, bioactivity and bioavailability. Food. Chem. x. 20, 100983. 10.1016/j.fochx.2023.100983 38144721 PMC10740132

[B14] Davinelli S. Bertoglio J. C. Zarrelli A. Pina R. Scapagnini G. (2015). A randomized clinical trial evaluating the efficacy of an anthocyanin-maqui berry extract (delphinol®) on oxidative stress biomarkers. J. Am. Coll. Nutr. 34, 28–33. 10.1080/07315724.2015.1080108 26400431

[B15] Diab M. Hamdi A. Al-Obeidat F. Hafez W. Cherrez-Ojeda I. Gador M. (2025). Discovery of drug transporter inhibitors tied to long noncoding RNA in resistant cancer cells; a computational model *-in silico-*study. Front. Immunol. 16, 1511029. 10.3389/fimmu.2025.1511029 40352931 PMC12061905

[B16] Ekundayo B. E. Ademola A. O. Ekundayo M. C. Deji-Oloruntoba O. O. Akomolafe E. F. Akomolafe B. K. (2025). Delphinidin: a multifaceted anthocyanidin with therapeutic potential in chronic diseases. J. Biochem. Mol. Toxicol. 39, e70655. 10.1002/jbt.70655 41423730

[B17] Favot L. Martin S. Keravis T. Andriantsitohaina R. Lugnier C. (2003). Involvement of cyclin-dependent pathway in the inhibitory effect of delphinidin on angiogenesis. Cardiovasc. Res. 59, 479–487. 10.1016/s0008-6363(03)00433-4 12909331

[B18] Feng R. Wang S. Y. Shi Y. H. Fan J. Yin X. M. (2010). Delphinidin induces necrosis in hepatocellular carcinoma cells in the presence of 3-methyladenine, an autophagy inhibitor. J. Agri. Food. Chem. 58, 3957–3964. 10.1021/jf9025458 20025272

[B19] Fleschhut J. Kratzer F. Rechkemmer G. Kulling S. E. (2006). Stability and biotransformation of various dietary anthocyanins *in vitro* . Eur. J. Nutr. 45, 7–18. 10.1007/s00394-005-0557-8 15834757

[B20] Fridrich D. Teller N. Esselen M. Pahlke G. Marko D. (2008). Comparison of delphinidin, quercetin and (-)-epigallocatechin-3-gallate as inhibitors of the EGFR and the ErbB2 receptor phosphorylation. Mole. Nutr. Food. Res. 52, 815–822. 10.1002/mnfr.200800026 18618485

[B21] Gottesman M. M. Robey R. W. Ambudkar S. V. (2023). New mechanisms of multidrug resistance: an introduction to the cancer drug resistance special collection. Cancer Drug Resist 6, 590–595. 10.20517/cdr.2023.86 37842242 PMC10571052

[B22] Gu P. Zhang M. Zhu J. He X. Yang D. (2022). Suppression of CDCA3 inhibits prostate cancer progression *via* NF-κB/cyclin D1 signaling inactivation and p21 accumulation. Oncol. Rep. 47, 42. 10.3892/or.2021.8253 34970697 PMC8759108

[B23] Hafeez B. B. Siddiqui I. A. Asim M. Malik A. Afaq F. Adhami V. M. (2008). A dietary anthocyanidin delphinidin induces apoptosis of human prostate cancer PC3 cells *in vitro* and *in vivo:* involvement of nuclear factor-kappaB signaling. Cancer Res. 68, 8564–8572. 10.1158/0008-5472.CAN-08-2232 18922932 PMC3149885

[B24] Han B. Peng X. Cheng D. Zhu Y. Du J. Li J. (2019). Delphinidin suppresses breast carcinogenesis through the HOTAIR/microRNA-34a axis. Cancer Sci. 110, 3089–3097. 10.1111/cas.14133 31325197 PMC6778627

[B25] He J. Giusti M. M. (2010). Anthocyanins: natural colorants with health-promoting properties. Annu. Rev. Food. Sci. Technol. 1, 163–187. 10.1146/annurev.food.080708.100754 22129334

[B26] Hou D. X. Ose T. Lin S. Harazoro K. Imamura I. Kubo M. (2003). Anthocyanidins induce apoptosis in human promyelocytic leukemia cells: structure-activity relationship and mechanisms involved. Int. J. Oncol. 23, 705–712. 12888907

[B27] Huang C. C. Hung C. H. Hung T. W. Lin Y. C. Wang C. J. Kao S. H. (2019). Dietary delphinidin inhibits human colorectal cancer metastasis associating with upregulation of miR-204-3p and suppression of the integrin/FAK axis. Sci. Rep. 9, 18954. 10.1038/s41598-019-55505-z 31831830 PMC6908670

[B28] Hwang M. K. Kang N. J. Heo Y. S. Lee K. W. Lee H. J. (2009). Fyn kinase is a direct molecular target of delphinidin for the inhibition of cyclooxygenase-2 expression induced by tumor necrosis factor-alpha. Biochem. Pharmacol. 77, 1213–1222. 10.1016/j.bcp.2008.12.021 19174152

[B29] Hyun K. H. Gil K. C. Kim S. G. Park S. Y. Hwang K. W. (2019). Delphinidin chloride and its hydrolytic metabolite gallic acid promote differentiation of regulatory T cells and have an anti-inflammatory effect on the allograft model. J. Food. Sci. 84, 920–930. 10.1111/1750-3841.14490 30977922

[B30] Im N. K. Jang W. J. Jeong C. H. Jeong G. S. (2014). Delphinidin suppresses PMA-Induced MMP-9 expression by blocking the NF-κB activation through MAPK signaling pathways in MCF-7 human breast carcinoma cells. J. Med. Food 17, 855–861. 10.1089/jmf.2013.3077 25000305

[B31] Jang C. H. Lee I. A. Ha Y. R. Lim J. Sung M. K. Lee S.-J. (2008). PGK1 induction by a hydrogen peroxide treatment is suppressed by antioxidants in human Colon carcinoma cells. Biosci. Biotechnol. Biochem. 72, 1799–1808. 10.1271/bbb.80079 18603805

[B32] Jara E. Hidalgo M. A. Hancke J. L. Hidalgo A. I. Brauchi S. Nuñez L. (2014). Delphinidin activates NFAT and induces IL-2 production through SOCE in T cells. Cell. biochem. Biophys. 68, 497–509. 10.1007/s12013-013-9728-z 23943055

[B33] Jeong M. H. Ko H. Jeon H. Sung G. J. Park S. Y. Jun W. J. (2016). Delphinidin induces apoptosis *via* cleaved HDAC3-mediated p53 acetylation and oligomerization in prostate cancer cells. Oncotarget 35, 56767–56780. 10.18632/oncotarget.10790 27462923 PMC5302952

[B34] Jiang S. Li H. Zhang L. Mu W. Zhang Y. Chen T. (2025). Generic diagramming platform (GDP): a comprehensive database of high-quality biomedical graphics. Nucleic. Acids Res. 53, D1670–D1676. 10.1093/nar/gkae973 39470721 PMC11701665

[B35] Kang N. J. Lee K. W. Kwon J. Y. Hwang M. K. Rogozin E. A. Heo Y. S. (2008). Delphinidin attenuates neoplastic transformation in JB6 Cl41 mouse epidermal cells by blocking raf/Mitogen-activated protein kinase kinase/extracellular signal-regulated kinase signaling. Cancer. Prev. Res. 1, 522–531. 10.1158/1940-6207.CAPR-08-0071 19139002 PMC2832759

[B36] Kang H. M. Park B. S. Kang H. K. Park H. R. Yu S. B. Kim I. R. (2018). Delphinidin induces apoptosis and inhibits epithelial-to-mesenchymal transition *via* the ERK/p38 MAPK-signaling pathway in human osteosarcoma cell lines. Environ. Toxicol. 33, 640–649. 10.1002/tox.22548 29451351 PMC5969316

[B37] Kang S. H. Bak D. H. Chung B. Y. Bai H. W. Kang B. S. (2020). Delphinidin enhances radio-therapeutic effects *via* autophagy induction and JNK/MAPK pathway activation in non-small cell lung cancer. Korean. J. Physiol. Pharmacol. 24, 413–422. 10.4196/kjpp.2020.24.5.413 32830148 PMC7445475

[B38] Karati D. Mukherjee S. Roy S. (2024). Emerging therapeutic strategies in cancer therapy by HDAC inhibition as the chemotherapeutic potent and epigenetic regulator. Med. Oncol. 41, 84. 10.1007/s12032-024-02303-x 38438564

[B39] Katsube N. Iwashita K. Tsushida T. Yamaki K. Kobori M. (2003). Induction of apoptosis in cancer cells by bilberry (Vaccinium myrtillus) and the anthocyanins. J. Agric. Food. Chem. 51, 68–75. 10.1021/jf025781x 12502387

[B40] Kausar H. Jeyabalan J. Aqil F. Chabba D. Sidana J. Singh I. P. (2012). Berry anthocyanidins synergistically suppress growth and invasive potential of human non-small-cell lung cancer cells. Cancer Lett. 325, 54–62. 10.1016/j.canlet.2012.05.029 22659736

[B41] Keravis T. Favot L. Abusnina A. A. Anton A. Justiniano H. Soleti R. (2015). Delphinidin inhibits tumor growth by acting on VEGF signalling in endothelial cells. PloS. One. 10, e0145291. 10.1371/journal.pone.0145291 26694325 PMC4687871

[B42] Khan H. Belwal T. Efferth T. Farooqi A. A. Sanches-Silva A. Vacca R. A. (2021). Targeting epigenetics in cancer: therapeutic potential of flavonoids. Crit. Rev. Food. Sci. Nutr. 61, 1616–1639. 10.1080/10408398.2020.1763910 32478608

[B43] Kim M. H. Jeong Y. J. Cho H. J. Hoe H. S. Park K. K. Park Y. Y. (2017). Delphinidin inhibits angiogenesis through the suppression of HIF-1α and VEGF expression in A549 lung cancer cells. Oncol. Rep. 37, 777–784. 10.3892/or.2016.5296 27959445

[B44] Ko H. Jeong M. H. Jeon H. Sung G. J. So Y. Kim I. (2015). Delphinidin sensitizes prostate cancer cells to TRAIL-Induced apoptosis, by inducing DR5 and causing caspase-mediated HDAC3 cleavage. Oncotarget 6, 9970–9984. 10.18632/oncotarget.3667 25991668 PMC4496411

[B45] Koss-Mikołajczyk I. Bartoszek A. (2023). Relationship between chemical structure and biological activity evaluated *in vitro* for six anthocyanidins Most commonly occurring in edible plants. Molecules 28, 6156. 10.3390/molecules28166156 37630408 PMC10458735

[B46] Kuo H. D. Wu R. Li S. Yang A. Y. Kong A. N. (2019). Anthocyanin delphinidin prevents neoplastic transformation of mouse skin JB6 P+ cells: epigenetic Re-activation of Nrf2-ARE pathway. AAPS. J. 21, 83. 10.1208/s12248-019-0355-5 31254216 PMC6669902

[B47] Kwon J. Y. Lee K. W. Kim J. E. Jung S. K. Kang N. J. Hwang M. K. (2009). Delphinidin suppresses ultraviolet B-induced cyclooxygenases-2 expression through inhibition of MAPKK4 and PI-3 kinase. Carcinogenesis 30, 1932–1940. 10.1093/carcin/bgp216 19776176 PMC2783004

[B48] Lamy S. Blanchette M. Michaud-Levesque J. Lafleur R. Durocher Y. Moghrabi A. (2006). Delphinidin, a dietary anthocyanidin, inhibits vascular endothelial growth factor receptor-2 phosphorylation. Carcinogenesis 27, 989–996. 10.1093/carcin/bgi279 16308314

[B49] Lamy S. Lafleur R. Bédard V. Moghrabi A. Barrette S. Gingras D. (2007). Anthocyanidins inhibit migration of glioblastoma cells: structure-activity relationship and involvement of the plasminolytic system. J. Cell. Biochem. 100, 100–111. 10.1002/jcb.21023 16823770

[B50] Lee W. Yun J. M. (2016). Suppression of β-catenin signaling pathway in human prostate cancer PC3 cells by delphinidin. J. Cancer. Prev. 21, 110–114. 10.15430/JCP.2016.21.2.110 27390740 PMC4933435

[B51] Lee D. Y. Park Y. J. Hwang S. C. Kim K. D. Moon D. K. Kim D. H. (2018). Cytotoxic effects of delphinidin in human osteosarcoma cells. Acta. Orthop. traumatol.turc. 52, 58–64. 10.1016/j.aott.2017.11.011 29290536 PMC6136320

[B52] Lim W. Song G. (2017). Inhibitory effects of delphinidin on the proliferation of ovarian cancer cells *via* PI3K/AKT and ERK 1/2 MAPK signal transduction. Oncol. Lett. 14, 810–818. 10.3892/ol.2017.6232 28693237 PMC5494655

[B53] Lim W. Jeong W. Song G. (2016). Delphinidin suppresses proliferation and migration of human ovarian clear cell carcinoma cells through blocking AKT and ERK1/2 MAPK signaling pathways. Mol. Cell. Endocrinol. 422, 172–181. 10.1016/j.mce.2015.12.013 26704080

[B54] Lim W. C. Kim H. Kim Y. J. Park S. H. Song J. H. Lee K. H. (2017). Delphinidin inhibits BDNF-Induced migration and invasion in SKOV3 ovarian cancer cells. Bioorg. Med. Chem. Lett. 27, 5337–5343. 10.1016/j.bmcl.2017.09.024 29122484

[B55] Lim W. C. Kim H. Ko H. (2019). Delphinidin inhibits epidermal growth factor-induced epithelial-to-mesenchymal transition in hepatocellular carcinoma cells. J. Cell. Biochem. 120, 9887–9899. 10.1002/jcb.28271 30537288

[B56] Lin B. W. Gong C. C. Song H. F. Cui Y. Y. (2017). Effects of anthocyanins on the prevention and treatment of cancer. Br. J. Pharmacol. 174, 1226–1243. 10.1111/bph.13627 27646173 PMC5429338

[B57] Liu X. Wang X. Jing N. Jiang G. Liu Z. (2020). Biostimulating gut microbiome with bilberry anthocyanin combo to enhance Anti-PD-L1 efficiency against murine Colon cancer. Microorganisms 8, 175. 10.3390/microorganisms8020175 31991820 PMC7074734

[B58] Lu Z. Kleeff J. Shrikhande S. Zimmermann T. Korc M. Friess H. (2000). Expression of the multidrug-resistance 1 (MDR1) gene and prognosis in human pancreatic cancer. Pancreas 21, 240–247. 10.1097/00006676-200010000-00004 11039467

[B59] Matsumoto H. Inaba H. Kishi M. Tominaga S. Hirayama M. Tsuda T. (2001). Orally administered delphinidin 3-rutinoside and cyanidin 3-rutinoside are directly absorbed in rats and humans and appear in the blood as the intact forms. J. Agric. Food Chem. 49, 1546–1551. 10.1021/jf001246q 11312894

[B60] Mazewski C. Kim M. S. Gonzalez de Mejia E. (2019). Anthocyanins, delphinidin-3-O-glucoside and cyanidin-3-O-glucoside, inhibit immune checkpoints in human colorectal cancer cells *in vitro* and *in silico* . Sci. Rep. 9, 11560. 10.1038/s41598-019-47903-0 31399602 PMC6689002

[B61] McGuire J. Taguchi T. Tombline G. Paige V. Janelsins M. Gilmore N. (2024). Hyaluronidase inhibitor delphinidin inhibits cancer metastasis. Sci. Rep. 14, 14958. 10.1038/s41598-024-64924-6 38942920 PMC11213947

[B62] Murata M. Marugame Y. Yamada S. Lin I. C. Yamashita S. Kumazoe M. (2025). Delphinidin upregulates microRNA-let-7b expression through family with sequence similarity 222 member B. Sci. Rep. 15, 29304. 10.1038/s41598-025-15588-3 40789939 PMC12340061

[B63] Norihito S. Wakana Y. Kenchi M. Hiroshi S. (2020). Ameliorating effects of delphinol®, anthocyanin standardized maqui berry extract, on skin brightness and redness in Japanese females: a randomized double-blind placebo-controlled pilot study. J. Cosmet. Dermatol. Sci. Appl. 10, 149–162. 10.4236/jcdsa.2020.104017

[B64] Ouanouki A. Lamy S. Annabi B. (2017). Anthocyanidins inhibit epithelial-mesenchymal transition through a TGFβ/Smad2 signaling pathway in glioblastoma cells. Mol. Carcinog. 56, 1088–1099. 10.1002/mc.22575 27649384

[B65] Ozbay T. Nahta R. (2011). Delphinidin inhibits HER2 and Erk1/2 signaling and suppresses growth of HER2-Overexpressing and triple negative breast cancer cell lines. Breast. Cancer. 5, 143–154. 10.4137/BCBCR.S7156 21792311 PMC3140266

[B66] Pal H. C. Sharma S. Strickland L. R. Agarwal J. Athar M. Elmets C. A. (2013). Delphinidin reduces cell proliferation and induces apoptosis of non-small-cell lung cancer cells by targeting EGFR/VEGFR2 signaling pathways. PloS One 8, e77270. 10.1371/journal.pone.0077270 24124611 PMC3790876

[B67] Peng J. Wu A. Yu X. Zhong Q. Deng X. Zhu Y. (2022). Combined network pharmacology and cytology experiments to identify potential anti-breast cancer targets and mechanisms of delphinidin. Nut. Cancer. 74, 2591–2606. 10.1080/01635581.2021.2012582 34875956

[B68] Pieńkowska N. Bartosz G. Furdak P. Sadowska-Bartosz I. (2021). Delphinidin increases the sensitivity of ovarian cancer cell lines to 3-Bromopyruvate. Int. J.Mol. Sci. 22, 709. 10.3390/ijms22020709 33445795 PMC7828231

[B69] Rahman M. M. Ichiyanagi T. Komiyama T. Hatano Y. Konishi T. (2006). Superoxide radical- and peroxynitrite-scavenging activity of anthocyanins; structure-activity relationship and their synergism. Free. Radic. Res. 40, 993–1002. 10.1080/10715760600815322 17015281

[B71] Rana J. N. Gul K. Mumtaz S. (2025). Isorhamnetin: reviewing recent developments in anticancer mechanisms and nanoformulation-driven delivery. Int. J. Mol. Sci. 26, 7381. 10.3390/ijms26157381 40806510 PMC12347436

[B72] Riaz S. Rasul A. Ahmad M. Asrar M. Hassan M. (2025). Pomegranate peel extract as 6-Phosphogluconate dehydrogenase (6PGD) inhibitor for treatment of breast cancer. Cell. biochem. Biophys. 83, 537–553. 10.1007/s12013-024-01485-5 39235507

[B73] Ribas A. Wolchok J. D. (2018). Cancer immunotherapy using checkpoint blockade. Science 359, 1350–1355. 10.1126/science.aar4060 29567705 PMC7391259

[B74] Sadowska-Bartosz I. Bartosz G. (2024). Antioxidant activity of anthocyanins and anthocyanidins: a critical review. Int. J. Mol. Sci. 25, 12001. 10.3390/ijms252212001 39596068 PMC11593439

[B75] Sharma A. Choi H. K. Kim Y. K. Lee H. J. (2021). Delphinidin and its glycosides' war on cancer: preclinical perspectives. Int. J. Mol. Sci. 22, 11500. 10.3390/ijms222111500 34768930 PMC8583959

[B76] Sinopoli A. Calogero G. Bartolotta A. (2019). Computational aspects of anthocyanidins and anthocyanins: a review. Food Chem. 297, 124898. 10.1016/j.foodchem.2019.05.172 31253334

[B77] Sun S. Xu K. Yan M. Cui J. Zhu K. Yang Y. (2023). Delphinidin induces autophagic flux blockage and apoptosis by inhibiting both multidrug resistance gene 1 and DEAD-Box helicase 17 expressions in liver cancer cells. J. Pharm. Pharmacol. 75, 253–263. 10.1093/jpp/rgac037 36179123

[B78] Takasawa R. Saeki K. Tao A. Yoshimori A. Uchiro H. Fujiwara M. (2010). Delphinidin, a dietary anthocyanidin in berry fruits, inhibits human glyoxalase I. Bioorg. Med. Chem. 18, 7029–7033. 10.1016/j.bmc.2010.08.012 20801663

[B79] Thiele W. Rothley M. Teller N. Jung N. Bulat B. Plaumann D. (2013). Delphinidin is a novel inhibitor of lymphangiogenesis but promotes mammary tumor growth and metastasis formation in syngeneic experimental rats. Carcinogenesis 34, 2804–2813. 10.1093/carcin/bgt291 23975834

[B80] Thoppil R. J. Bhatia D. Barnes K. F. Haznagy-Radnai E. Hohmann J. Darvesh A. S. (2012). Black currant anthocyanins abrogate oxidative stress through Nrf2-mediated antioxidant mechanisms in a rat model of hepatocellular carcinoma. Curr. Cancer. Drug. Targets 12, 1244–1257. 10.2174/156800912803987968 22873220

[B81] Wan P. Li X. Guo S. Zhao X. (2024). Combination effect of flavonoids attenuates lung cancer cell proliferation by inhibiting the STAT3 and FAK signaling pathway. Open. Life. Sci. 19, 20220977. 10.1515/biol-2022-0977 39588118 PMC11588013

[B82] Wang L. S. Stoner G. D. (2008). Anthocyanins and their role in cancer prevention. Cancer Lett. 269, 281–290. 10.1016/j.canlet.2008.05.020 18571839 PMC2582525

[B83] Wang X. Kang Y. Li J. Jing L. Zhang Y. (2021). Antiproliferative and apoptosis inducing inducing effect of delphinidin against human bladder cancer cell line. Pharmacogn. Mag. 17, 101–105. 10.4103/pm.pm_548_19

[B84] Wang M. Liu K. Bu H. Cong H. Dong G. Xu N. (2022). Purple sweet potato delphinidin-3-rutin represses glioma proliferation by inducing miR-20b-5p/Atg7-dependent cytostatic autophagy. Mol. Ther. Oncolytics 26, 314–329. 10.1016/j.omto.2022.07.007 36090477 PMC9420429

[B103] Wang X. Li J. Chen R. Li T. Chen M. (2023). Active ingredients from chinese medicine for combination cancer therapy. Int. J. Biol. Sci. 19, 3499–3525. 10.7150/ijbs.77720 37497002 PMC10367560

[B85] Wang D. Lv L. Du J. Tian K. Chen Y. Chen M. (2024). TRIM16 and PRC1 are involved in pancreatic cancer progression and targeted by delphinidin. Chem. Biol. Drug. Des. 104, e70026. 10.1111/cbdd.70026 39635962

[B86] Watson R. R. Schönlau F. (2015). Nutraceutical and antioxidant effects of a delphinidin-rich maqui berry extract delphinol®: a review. Minerva. Cardioangiol. 63, 1–12. PMID:25892567. 25892567

[B87] Winkel-Shirley B. (2001). Flavonoid biosynthesis. A colorful model for genetics, biochemistry, cell biology, and biotechnology. Plant Physiol. 126, 485–493. 10.1104/pp.126.2.485 11402179 PMC1540115

[B88] Wu A. Zhu Y. Han B. Peng J. Deng X. Chen W. (2021). Delphinidin induces cell cycle arrest and apoptosis in HER-2 positive breast cancer cell lines by regulating the NF-κB and MAPK signaling pathways. Oncol. Lett. 22, 832. 10.3892/ol.2021.13093 34712357 PMC8548810

[B89] Xue H. Zhao J. Wang Y. Shi Z. Xie K. Liao X. (2024). Factors affecting the stability of anthocyanins and strategies for improving their stability: a review. Food. Chem. x. 24, 101883. 10.1016/j.fochx.2024.101883 39444439 PMC11497485

[B90] Yang F. Lee G. Fan Y. (2024). Navigating tumor angiogenesis: therapeutic perspectives and myeloid cell regulation mechanism. Angiogenesis 27, 333–349. 10.1007/s10456-024-09913-z 38580870 PMC11303583

[B91] Yeh C. T. Yen G. C. (2005). Induction of apoptosis by the anthocyanidins through regulation of Bcl-2 gene and activation of c-Jun N-terminal kinase Cascade in hepatoma cells. J. Agric. Food. Chem. 53, 1740–1749. 10.1021/jf048955e 15740068

[B92] Yoshino Y. Yuan B. Okusumi S. Aoyama R. Murota R. Kikuchi H. (2018). Enhanced cytotoxic effects of arsenite in combination with anthocyanidin compound, delphinidin, against a human leukemia cell line, HL-60. Chem. Biol. Interact. 294, 9–17. 10.1016/j.cbi.2018.08.008 30125548

[B93] Yu X. Song X. Yan J. Xiong Z. Zheng L. Luo Y. (2024). Inhibition of triple-negative breast cancer growth *via* delphinidin-mediated suppression of the JAK2/STAT3/PD-L1 pathway. Food. Nutr. Res. 68, 10974. 10.29219/fnr.v68.10974 39781272 PMC11708511

[B94] Yuan B. Okusumi S. Yoshino Y. Moriyama C. Tanaka S. Hirano T. (2015). Delphinidin induces cytotoxicity and potentiates cytocidal effect in combination with arsenite in an acute promyelocytic leukemia NB4 cell line. Oncol. Rep. 34, 431–438. 10.3892/or.2015.3963 25954945

[B95] Yücetepe M. Tuğba Özaslan Z. Karakuş M. Ş. Akalan M. Karaaslan A. Karaaslan M. (2024). Unveiling the multifaceted world of anthocyanins: biosynthesis pathway, natural sources, extraction methods, copigmentation, encapsulation techniques, and future food applications. Food Res. Int. 187, 114437. 10.1016/j.foodres.2024.114437 38763684

[B96] Yun J. M. Afaq F. Khan N. Mukhtar H. (2009). Delphinidin, an anthocyanidin in pigmented fruits and vegetables, induces apoptosis and cell cycle arrest in human Colon cancer HCT116 cells. Mol. Carcinog. 48, 260–270. 10.1002/mc.20477 18729103 PMC2946888

[B97] Zafar A. Khan M. J. Abu J. Naeem A. (2024). Revolutionizing cancer care strategies: immunotherapy, gene therapy, and molecular targeted therapy. Mol. Biol. Rep. 51, 219. 10.1007/s11033-023-09096-8 38281269 PMC10822809

[B98] Zhang Y. Vareed S. K. Nair M. G. (2005). Human tumor cell growth inhibition by nontoxic anthocyanidins, the pigments in fruits and vegetables. Life. Sci. 76, 1465–1472. 10.1016/j.lfs.2004.08.025 15680311

[B99] Zhang Z. Pan Y. Zhao Y. Ren M. Li Y. Lu G. (2021). Delphinidin modulates JAK/STAT3 and MAPKinase signaling to induce apoptosis in HCT116 cells. Environ. Toxicol. 36, 1557–1566. 10.1002/tox.23152 33955636

[B100] Zhao J. (2015). Flavonoid transport mechanisms: how to go, and with whom. Trends. Plant. Sci. 20, 576–585. 10.1016/j.tplants.2015.06.007 26205169

[B101] Zhao J. Zhang L. Zhao Y. Wu N. Zhang X. Guo R. (2023). Long noncoding RNA HOTAIR promotes breast cancer development through the lncRNA HOTAIR/miR-1/GOLPH3 axis. Clin. Transl. Oncol. 25, 3420–3430. 10.1007/s12094-023-03197-3 37099061

[B102] Zou J. Y. Chen Q. L. Luo X. C. Damdinjav D. Abdelmohsen U. R. Li H. Y. (2024). Natural products reverse cancer multidrug resistance. Front. Pharmacol. 15, 1348076. 10.3389/fphar.2024.1348076 38572428 PMC10988293

